# 5′-UTR and 3′-UTR Regulation of MICB Expression in Human Cancer Cells by Novel microRNAs

**DOI:** 10.3390/genes8090213

**Published:** 2017-08-29

**Authors:** Wipaporn Wongfieng, Amonrat Jumnainsong, Yaovalux Chamgramol, Banchob Sripa, Chanvit Leelayuwat

**Affiliations:** 1 Biomedical Sciences Program, Graduates School of Khon Kaen University, Khon Kaen 40002, Thailand; wongfieng@hotmail.com; 2 Department of Clinical Immunology and Transfusion Sciences, Faculty of Associated Medical Sciences, Khon Kaen University, Khon Kaen 40002, Thailand; amonrat@kku.ac.th; 3 Liver Fluke and Cholangiocarcinoma Research Center, Faculty of Medicine, Khon Kaen University, Khon Kaen 40002, Thailand; cyaova@yahoo.com (Y.C.); banchob@kku.ac.th (B.S.); 4 The Centre for Research and Development of Medical Diagnostic Laboratories (CMDL), Faculty of Associated Medical Sciences, Khon Kaen University, Khon Kaen 40002, Thailand; 5 Department of Pathology, Faculty of Medicine, Khon Kaen University, Khon Kaen 40002, Thailand

**Keywords:** natural killer group 2, member D (NKG2D) ligands, natural killer group 2, member D (NKG2D), major histocompatibility complex (MHC) class I chain related protein B (MICB), microRNA, 5′-UTR regulation

## Abstract

The treatment of cancer through the induction of natural killer group 2, member D (NKG2D) ligands is of interest, but understanding of mechanisms controlling expression of individual ligand is limited. The major histocompatibility complex (MHC) class I chain related protein B (MICB) is a member of NKG2D ligands. We aimed to investigate the role of 3′-untranslated (3′-UTR) and 5′-untranslated regions (5′-UTR) in post-transcriptional regulation of MICB. Nine novel microRNAs (miRNAs) predicted to interact with 3′-UTR and 5′-UTR using TargetScan, RNAhybrid and miBridge were identified. Their regulation of 3′-UTR, 5′-UTR and both 3′- and 5′-UTR sequences of MICB were indicated by the reduction of luciferase activities of luciferase reporter constructs. Mutations of miRNA binding sites at 3′- and 5′-UTRs resulted in increased luciferase activities confirming the regulation of nine candidate miRNAs. In addition, overexpression of candidate miRNAs also down-regulated the expression of reporter constructs. Consequently, the overexpression and inhibition of candidate miRNAs lead to the decreased and increased. MICB protein expressions on the cells tested, respectively. This study has identified a new role of miRNAs in regulation of MICB expression via both 3′-UTR and 5′-UTR sequences applicable for cancer immunotherapy.

## 1. Introduction

The natural killer group 2, member D (NKG2D) system consists of the NKG2D receptor expressed on natural killer (NK) cells, cluster of differentiation 8+ (CD8+) thymus (T)-cells and natural killer thymus (NKT) cells and NKG2D ligands (NKG2D-Ls), frequently upregulated in stressed cells such as viral or bacterial infected cells and tumor cells [1]. However, they are restrictively expressed on normal cells [1,2]. Binding of NKG2D to its ligands activates immune cells bearing this receptor and promotes cytotoxicity of the cells expressing these ligands [1,3]. The striking feature of NKG2D system is that a single receptor recognizes multiple families of polymorphic ligands [4,5]. Currently there are eight molecules of human NKG2D-Ls classified into two families; MHC class I chain related protein A and B (MICA and MICB) family, and UL16-binding proteins (ULBP1-6) or retinoic acid early inducible transcript-1 (RAET1) [6,7]. The mechanisms involved NKG2D-L expression present an important role in response to stressed cells through induction of immune function and also area promising therapeutic target for the responding of immune responses against tumor cells. For therapeutic purposes, how the expression of NKG2D-L is controlled, needs to be thoroughly studied. Tight regulation of NKG2D-L expression is essential. To avoid autoimmunity, normal cells should not express NKG2D-Ls but rapid up-regulation is required upon stress, infection or cancer transformation [8]. Surprisingly, numerous reports demonstrated that different cells and tissues expressed messenger RNAs (mRNAs) for NKG2D-Ls but lacked any expression of the corresponding proteins. These observations suggest that at least some of the NKG2D-Ls are also regulated at post-transcriptional level [9].

MicroRNAs (miRNA) are small, endogenous and non-coding RNAs (typically comprised of 19 to 25 nucleotides (nt)), which exert their negatively post-transcriptional regulation by targeting 3′-UTRs of the target mRNAs leading to mRNA degradation or translation inhibition [10,11]. miRNA can simultaneously target both 5′- and 3′-untranslated regions (UTRs) leading to enhanced regulations [12]. Evidently, gene regulation via miRNAs is a fundamental mechanism of post-transcriptional regulation having various functions [13]. In cancer, aberrant miRNA expression is correlated with clinically relevant characteristics [14]. miRNAs are also important in regulating the immune response including cell development, differentiation and modulation of both innate and adaptive immune responses [15]. Recently, several groups have identified endogenous cellular miRNAs that could control MICA, MICB, ULBP1 and ULBP2 expressions by targeting their 3′-UTRs [16,17,18,19]. However, previous studies reported that miRNA could violate the rule of 3′-UTR dependent miRNA function. A luciferase reporter gene assay and computational predictions indicated that miRNAs could also bind to 5′-UTR [20,21]. miBridge [12], an online accessible collection of miRNA target containing miRNA binding sites in both UTRs, has been developed. Combinatory interaction not only reduced the number of predicted miRNAs but also showed further repression of target mRNAs. Based on discovery of miRNAs regulating NKG2D-Ls and 5′-UTR mediating miRNA function, it is possible that miRNA based regulation of both UTRs of NKG2D-Ls exists.

In this study, functional analyses of miRNAs predicted to regulate MICB at both UTRs have been evaluated in cancer cell lines. The finding of this study may reinforce the role of miRNAs in modulating the NKG2D-L levels applicable for cancer immunotherapeutic approaches.

## 2. Materials and Methods

### 2.1. Bioinformatic Prediction

Both 3′-and 5′-UTR sequences of MICB are available from Gene Bank via accession numbers; NM_005931 position at 1–116 for 5′-UTR and position at 1269–2497 for 3′-UTR. All human miRNAs can be downloaded from miRbase [22]. The miRNAs simultaneously binding to both UTRs of MICB is predicted as described in Lee et al. [12] and interactions with its 3′-UTR were analyzed by three computational algorithms (TargetScan [23], miRanda [24] and RNAhybrid [25]).

### 2.2. Real Time Quantitative PCR (qRT-Polymerase Chain Reaction)

The cells were isolated for total miRNA and mRNA by using TRIZOL (Invitrogen, Carlsbad, CA, USA). Then, the RNA samples were reverse-transcribed to complementary DNA (cDNA) by using the Moloney Murine Leukemia virus (MMLV) reverse transcriptase (Promega, Madison, WI, USA), oligo(dt) primer, gene specific primers and stem-loop reverse transcript (RT) primers [26,27] (for reverse-transcription of mature miRNA).

MICB DNA was amplified with specific primers [28] (Supplementary Table S1) and the QuantiFast SYBR Green PCR kit (QIAGEN, Germantown, MD, USA) and analyzed on an ABI PRISM 7500 Real-Time PCR system (Applied Biosystems, Waltham, MA, USA). Relative expression was calculated as: 2^ΔCt^, where ΔCT = CT_NKG2DL_− CT_GADPH_. For mature miRNA, DNA was amplified with forward primers specific to miRNAs and a universal reverse primer [27] (Supplementary Table S2). Relative expression was calculated as: 2^ΔCt^, where ΔCT = CT_givenmiRNA_ − CT_miR__-16_.

### 2.3. Cell Lines

One immortalized cholangiocyte (MMNK1), two CCA cell lines (KKU-213 and KKU-214) (Khon Kaen University, Khon Kaen, Thailand) and three cervical cancer cell lines (HeLa, ShiHa and Caski) were used in this study. All cell lines were grown in Dulbecco’s Modified Eagle’s Medium (DMEM) (Invitrogen) supplemented with 10% fetal bovine serum and 1% penicillin-streptomycin (Sigma, St. Louis, MO, USA) and were maintained at 37 °C in 5% CO_2_ air atmosphere.

### 2.4. Plasmid Constructs

The Firefly luciferase vector and the *Renilla* vector were obtained from Texas University, USA. The wild-type 3′-UTR of MICB was inserted into BamH I and NotI sites downstream of *pcDNA3.1(zeo)+Pp* luciferase gene (pMICB_3U) and the wild-type 5′-UTR of MICB was inserted into HindIII and NheI sites upstream of *pcDNA3.1(zeo)+Pp* luciferase gene (pMICB_5U) and both the wild-type 3′- and 5′-UTRs were inserted downstream and upstream of *pcDNA3.1(zeo)+Pp* luciferase gene, respectively (pMICB_3U_5U). Site specific mutation of all candidate miRNAs and known miRNA binding sites were mutated by PCR directed mutagenesis (all primers are shown in Supplementary Tables S3 and S4). All plasmid constructs and related information are listed in Table 1. For the miRNA mimic vector constructs and the pre-miRNA hairpin templates were designed and ordered from Bio Basic Inc. (Markham, ON, Canada). Complementary pre-miRNA hairpins were amplified by PCR. The PCR condition and miRNA mimic vector constructions were performed according to Dow et al. [29]. The inserts and their proper orientations were confirmed by DNA sequencing (Macrogen, Seoul, Korea).

### 2.5. Luciferase Reporter Assay

The 293T cells were transfected with 50 ng of pcDNA3.1(zeo)+Pp luciferase reporter vector or *pcDNA3.1(zeo)+* *Pp* construct containing either wild-type or mutated 3′-UTR and 5′-UTR and were co-transfected with 5 ng pRL-SV 40 vector (*Renilla* luciferase) (Promega). Twenty-four hours after transfection, Firefly and *Renilla* luciferase activities were measured consecutively using the dual-luciferase reporter assays (Promega) by the GloMax^®^ 20/20 luminometer machine (Promega). To give relative expression, the relative luciferase values were calculated via several normalizations First, Firefly luciferase activity (Pp) from pcDNA3.1(zeo)+Pp construct containing either wild-type or mutated of 3′-UTR and 5′-UTR or pcDNA3.1(zeo)+Pp (empty vector) were normalized with *Renilla* luciferase activity (Rr). Second, the Pp/Rr values of luciferase reporter plasmids were subsequently normalized to Pp/Rr values of the cytomegalovirus (CMV) promoter from pcDNA3.1-Zeo (+)Pp, yielding relative Pp/Rr values (y axis). The values of pcDNA3.1-Zeo (+)Pp were set as 1. Each Firefly plasmid was tested in each experiment in triplicates. Student’s *t*-test was used to compare the effect of different reporter vectors on average luciferase activities [16,30].

### 2.6. miRNA Overexpression

HeLa cells were seeded at 5 × 10^4^ cells/well in 24-well plates and cultured until 70–80% confluent cell growth and transiently transfected with 5 μg of each mimic miRNA vector by using the FuGene^®^ HD transfection reagent (Roche, Basel, Switzerland) according to the manufacturer’s instructions. The cells were harvested 24 h after transfection, and then miRNAs and MICB protein expression levels were detected by qRT-PCR and flow cytometry.

### 2.7. miRNA Inhibition

KKU-214 cells were seeded at 5 × 10^4^ cells/well in 24-well plates and cultured until 70–80% confluent cell growth then transfected with 500 nM of anti-miR-320a, anti-miR-940 or the scramble control (mirVana^TM^, Applied Biosystems) by using FuGene^®^ HD transfection reagent (Roche) according to the manufacturer’s instructions. The cells were harvested 24 h after transfection and measured for MICB expression by flow cytometry.

### 2.8. Flow Cytometry

The cell lines were stained with 10 mg/mL of allophycocyanin (APC)-labeled anti- human MICB mouse antibody or isotype control (R&D Biosystems, Minneapolis, MN, USA) for 30 min on ice. Then the cells were washed with 1% bovine serum albumin in 1Xphosphate buffered saline (PBS) and finally analyzed on the BD FACSCanto II flow cytometer (Becton Dickinson, San Jose, CA, USA). The MICB expression levels on cell lines were determined by the mean fluorescence intensity (MFI) calculated by the MFI of cells stained with an anti-MICB antibody divided by the MFI of cells stained with the isotype antibody. At least 10,000 events were acquired and the analysis was performed using the FACS Diva software (Becton Dickinson).

### 2.9. Statistical Analysis

All data were presented as the means ± standard error of mean (S.E.M). Student’s *t*-test was used to determine statistically significant differences between the groups by using the GraphPad Pro. Prism5.0 (GraphPad, San Diego, CA, USA). *p*-values < 0.05 were determined as statistical significance. Data were representatives of at least three independent experiments.

## 3. Results

### 3.1. Bioinformatic Predictions of Candidate miRNAs

To identify candidate miRNAs that potentially downregulated MICB expression, three different computational algorithms were used to predict the miRNAs simultaneously binding to both 3′-UTR and 5′-UTR of MICB. They were TargetScan [23], RNAhybrid (BiBiserv2) [25] and miBridge [12]. All three programs identified nine miRNAs that could bind both 3′-UTR and 5′-UTR sequences of MICB (Table 2). Evidently, these miRNAs have not been reported to regulate MICB expressions.

**Figure 1:**
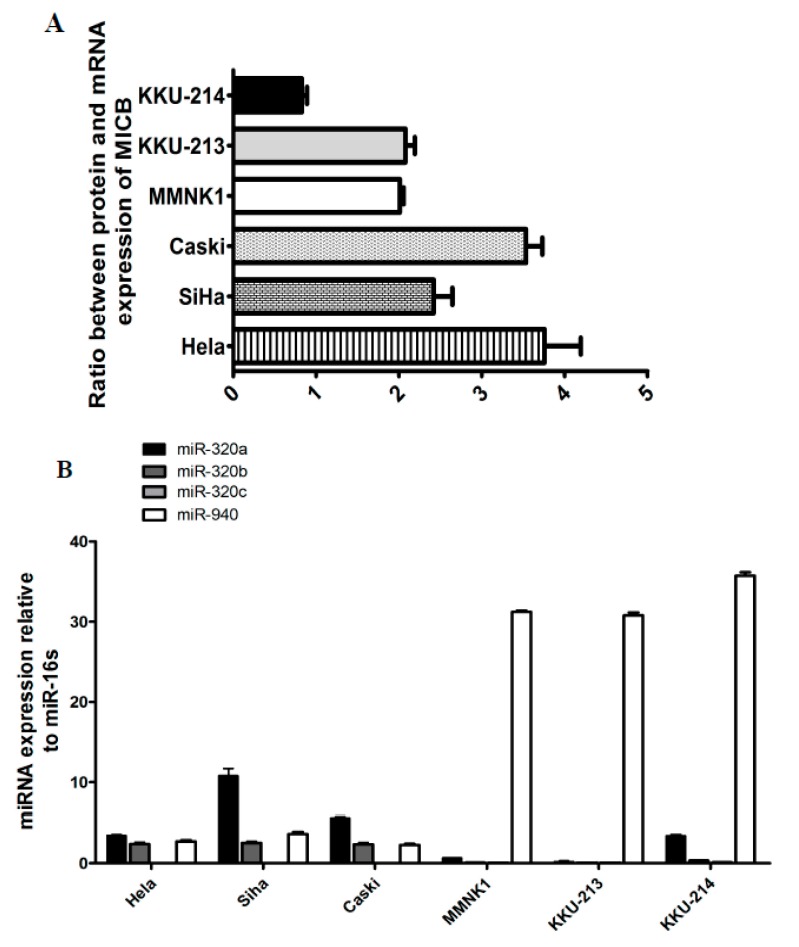
The endogenous expression of protein and messenger RNA (mRNA) of MHC class I chain related protein B (MICB) as well as candidate microRNAs (miRNAs) in various human cancer cell lines. (**A**) The protein expression levels of MICB were divided by mRNA expression levels of MICB to show ratio between protein and mRNA expression; (**B**) Endogenous expression levels of nine candidate miRNAs were measured in human cancer cell lines by quantitative real-time polymerase chain reaction (qRT-PCR) (candidate miRNAs that were not detected are not shown). The expression levels were normalized to miR-16. The results are depicted as mean ± standard error of mean (S.E.M). Relative expression was calculated as: 2^ΔCt^, where ΔCT = CT_givenmiRNA_ − CT_miR__-__16_.

To determine whether MICB expression on various cells tested were regulated at the post-transcriptional level, we evaluated miRNA, target mRNA co-expressions and protein expressions. Firstly, we detected MICB mRNA expression levels compared with MICB surface protein expression levels on human cancer cell lines (cervical cancer and cholangiocarcinoma or CCA cell lines). The cells were investigated for MICB surface protein expressions by flow cytometry and mRNA expression by qRT-PCR. The mRNA of MICB in KKU-214 (CCA) were higher than the other cancer cell lines except the immortal cholangiocytes (Supplementary Materials Figure S1) but the protein expression levels were lower (Supplementary Materials Figure S2). Consequently, the ratio of MICB mRNAs and proteins were lowest in KKU-214 (Figure 1A). The inverse expression between MICB mRNAs and proteins suggested that a post-transcriptional regulation might exist. Then, to assess whether the candidate miRNAs were expressed in different cancer cell lines, the stem-loop RT-qPCR was employed to detect candidate miRNAs in HeLa, Caski, SiHa, MMNK1, KKU-213 and KKU-214. MiR-320a, miR-320b and miR-940 were expressed in all cell lines whereas miR-320c was only expressed in KKU-214 (Figure 1B). The other candidate miRNAs were not detected (data not shown). It is possible that these candidate miRNAs present in these cell lines could regulate MICB expressions.

### 3.2. Down-Regulation of MICB via 3′-UTR and 5′-UTR Sequences

**Figure 2:**
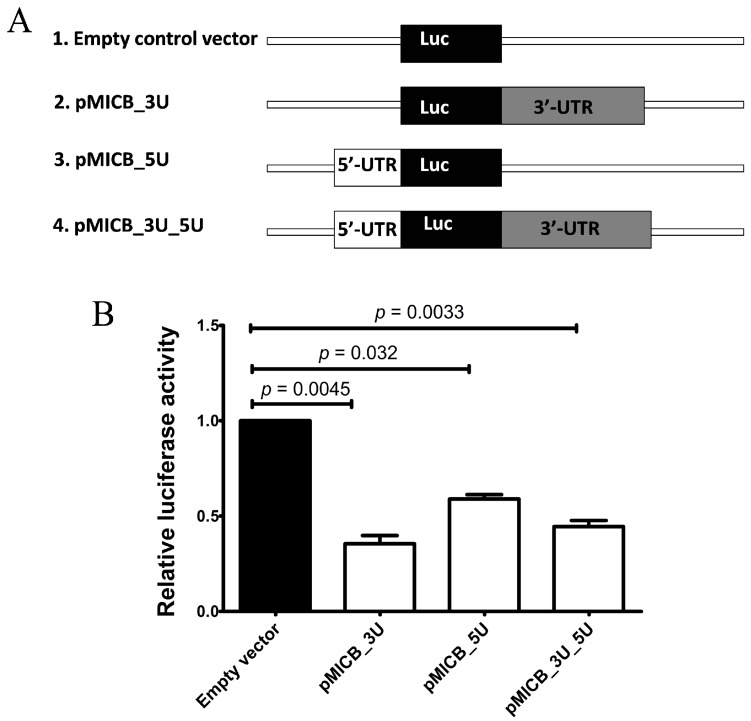
The effect of 3’-and 5’-untranslated regions (UTRs) of MICB on luciferase activity. (**A**) The constructs used for transfection in these experiments are represented. Luciferase gene alone is an empty control vector. Plasmids containing3′-UTR, 5′-UTR and both 3′- and 5′-UTR inserts are abbreviated with pMICB_3U, pMICB_5U and pMICB_3U_5U, respectively; (**B**) Effect of 3′-UTR and 5′-UTR of MICB on luciferase expression when these vectors were transfected to 239T cells are shown. Firefly luciferase activity was normalized with *Renilla* luciferase activity to give relative expression and subsequently normalized to the activity of control reporter. Results are depicted as mean ± S.E.M, *n* = 3 experiments.

To investigate whether both 3′-UTR and 5′-UTR of MICB were involved in down regulation of MICB, the 293T cells were used in luciferase reporter assay. The 293T cells were transfected with luciferase reporter constructs containing the 3′-UTR, 5′-UTR or constructs containing both 3′- and 5′-UTR of MICB and co-transfected with pRL-SV40. After transfection was completed, the cells were harvested and luciferase activities were measured using the dual-luciferase reporter assay. Luciferase activity of reporter constructs (Figure 2A) containing 3′-UTR (pMICB_3U), 5′-UTR (pMICB_5U) and both UTRs (pMICB_3U_5U) (details of plasmids are showed in Table 2) were significantly reduced to 60%, 30% and 50%, respectively, compared to the empty control vector (Figure 2B). The data indicated that 3′-UTR and 5′-UTR of MICB contained regulatory element(s). However, when both 3′- and 5′-UTR were included, the luciferase activities were no further reduced than with the 3′-UTR alone. Thus, the mechanism of regulated MICB expression may not be a miBridge type according to the study of Lee et al. [12].

### 3.3. Candidate miRNAs Directly Interacted with 3-′UTR of MICB

**Figure 3:**
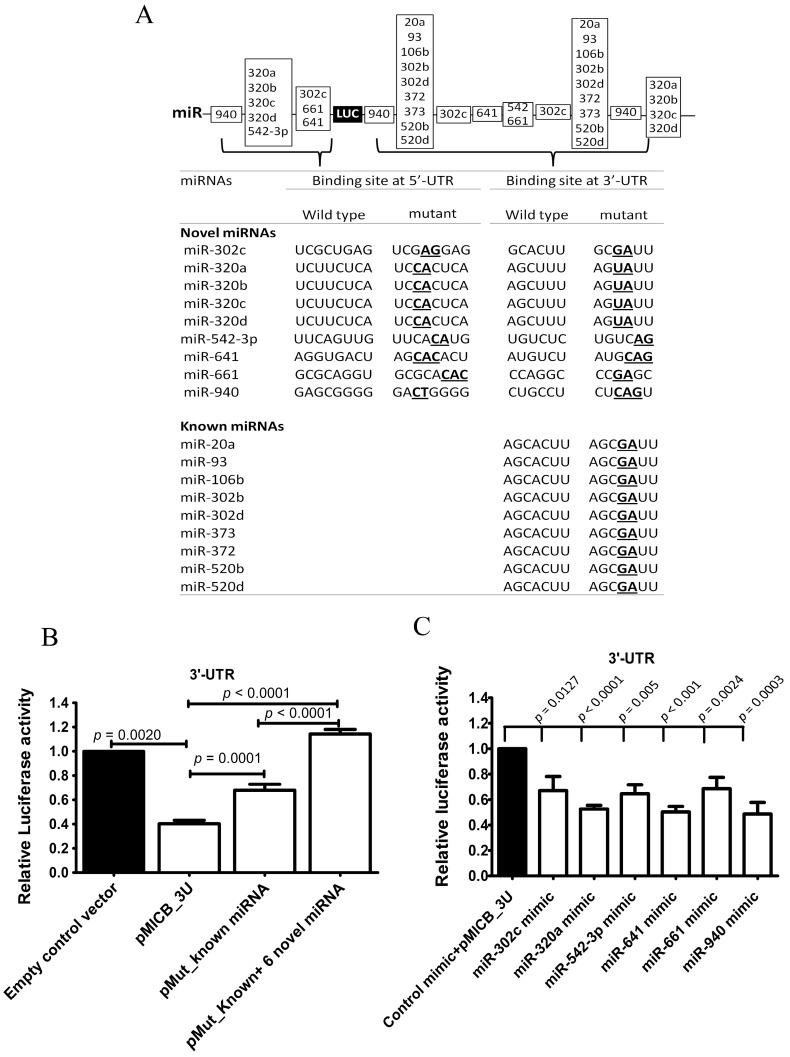

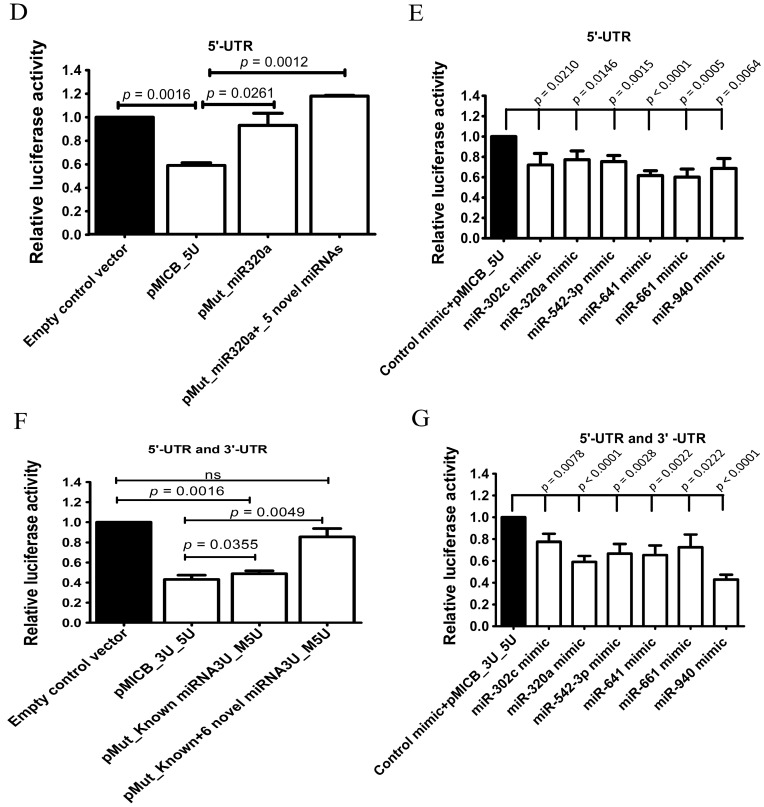
Candidate miRNAs directly interacted to 3’-UTR and 5’-UTR of MICB. (**A**) The predicted binding sites of candidate miRNAs were mutated by PCR directed mutagenesis and were confirmed by DNA sequencing as shown; (**B**) Relative luciferase activity after transiently transfected with a luciferase reporter fused to a 3′-UTR of MICB (pMICB_3U), the mutated 3′-UTR of MICB for the binding sites of known miRNAs (pMut_Known miRNAs_3U), the mutated 3′-UTR of MICB for the binding sites of both known miRNAs and novel miRNAs (pMut_Known+6 Novel miRNAs_3U) or luciferase gene alone (empty control vector) into 293T cells; (**C**) Relative luciferase activity after overexpressing candidate miRNAs in 293T cell and co-transfected with a luciferase reporter fused to a 3′-UTR of MICB (pMICB_3U); (**D**) Relative luciferase activity after transiently transfected with a luciferase reporter fused to a 5′-UTR of MICB (pMICB_5U), a mutated binding site of only miR-320a (pMut_320a), a mutated binding sites of all candidate miRNAs (pMut_miR320a+5 Novel miRNAs) or luciferase gene alone (empty control vector) into 293T cells; (**E**) Relative luciferase activity after overexpressing candidate miRNAs in 293T cell and co-transfected with a luciferase reporter fused to a 5′-UTR of MICB (pMICB_5U); (**F**) Mutated binding sites of only candidate miRNAs on 5′-UTR (pMut_Known _3U_M5U) and, mutated both binding sites of known and candidate miRNAs on 3′-UTR and mutated binding sites of only candidate miRNAs on 5′-UTR (pMut_Known+6 Novel miRNAs_3U_M5U); (**G**) Relative luciferase activity after overexpressing candidate miRNAs in 293T cell and co-transfected with a luciferase reporter fused to both 3′- and 5′-UTR (pMICB_3U_5U). Firefly luciferase activity was normalized with *Renilla* luciferase activity and subsequently normalized to the activity of control reporter. Results are depicted as mean ± S.E.M, *n* = 3 experiments.

To investigate the possible mechanism that regulated MICB expression via 3′-UTR of MICB, site directed mutagenesis was performed using PCR. The wild type 3′-UTR of MICB and the mutated miRNA binding sites on 3′-UTR of MICB were cloned into luciferase reporter constructs. Because of the previous study of Stern-Ginossaret al. reported that miRNAs (miR-20a, miR-93 and miR-106b) could downregulate MICA and MICB expression [16], two types of reporter mutants were generated. One type of constructs contained the mutated binding sites of both known miRNAs (miR-20a, miR-93 and miR-106b) and nine novel miRNAs (our candidate miRNAs, miR-320c, miR-320a, miR-320b, miR-320c, miR-320d, miR-542-3p, miR-641, miR-661 and miR-940) and another type contained only the mutated binding sites of known miRNAs as a positive control (Figure 3A). These reporter constructs were transfected into 293T cells. Luciferase activities of the construct containing mutated both known and novel miRNAs binding sites (pMut_Known+6 Novel miRNAs) were strongly and significantly increased. Accordingly, the construct containing only mutated known miRNAs binding sites (pMut_Known miRNAs) was also significantly increased compared to the construct containing wild-type 3′-UTR-MICB (pMICB_3U) (Figure 3B) but at a lesser extent. The results indicated that candidate miRNAs existing in 293T cells could actually regulate via 3′-UTR of MICB.

In addition to mutation, overexpression of miRNA experiments confirmed the role of candidate miRNAs in MICB regulation. Each candidate miRNA was overexpressed in 293T cells and validated whether highly expressed levels of these candidate miRNAs could lead to the reduction of luciferase activity. The overexpressing miRNA mimic vectors (miR-302c, miR-320a, miR-542-3p, miR-641, miR-661 and miR-940 mimics) were transiently transfected into 293T cells and were co-transfected with luciferase reporter constructs containing wild-type 3-′UTR of MICB (pMICB_3U). After the transfections were completed, the cells were measured for miRNA expression levels. The overexpression experiments of miRNAs were successful with higher expression level of the mimic miRNAs than the endogenous miRNAs (Supplementary Figure S3A) and then the cells were measured for luciferase activities using the dual-luciferase reporter assay. Expectedly, for the wild-type 3′-UTR construct, the luciferase activities were strongly and significantly decreased at the high levels of miR-320a, miR542-3p, miR-641 and miR-940 and a moderately but significant decreased in the presence of miR-302c and miR-661 compared with the control mimic vector (Figure 3C). These results indicated that miRNA candidates (miR-320a, miR-542-3p, miR641, miR-940, miR-302c and miR-661) interacted with a predicted binding site on 3′-UTR of MICB.

### 3.4. Candidate miRNAs Directly Interacted to 5′-UTR of MICB

The 5′-UTR of MICB may be involved in MICB regulation as previously shown (Figure 2B) but not via mechanism of the miBridge model system. Therefore, we aimed to investigate possible mechanism that regulated MICB expression whether 5′-UTR of MICB was directly and independently targeted by candidate miRNAs. Luciferase reporter constructs containing 126 bp of wild type 5′-UTR-MICB at upstream of luciferase genes (pMICB_5U) and a mutated binding site of only miR-320a (pMut_320a) because the endogenous miR-320a in 293T cell lines are higher than other miRNA (Supplementary Figure S3B) and mutated binding sites of all candidate miRNAs (pMut_miR320a+5 Novel miRNAs) on 5′-UTR-MICB (Figure 3A) were firstly generated. Interestingly, the luciferase activities were moderately but significantly recovered and were strongly and significantly recovered in the construct containing a mutated binding site of only miR-320a and the construct containing mutated binding sites of all candidate miRNAs, respectively, when were compared with a construct containing wild-type 5′-UTR-MICB (Figure 3D). These data suggested that not only miR-320a promote the suppression by directly interacted at a predicted binding site in 5′-UTR but also other candidate miRNAs. Consequently, to confirm hypothesis of these studies we constructed plasmids to overexpress candidate miRNAs (miR-302c, miR-320a, miR-542-3p, miR-641, miR-661 and miR-940 mimic) and then these mimic miRNAs or control mimic plasmids were co-transfected with reporter construct containing wild-type 5′-UTR (pMICB_5U) into 293T cells. After overexpression, the mimic miRNAs were present at higher expression levels than the endogenous miRNAs, especially miR-641 and miR-661 (Supplementary Figure S3C). Accordingly, the luciferase activities were strongly and significantly decreased with miR-641 and miR-661, a moderately but significantly decreased with miR-542-3p and miR-940 and a slightly but also significantly decreased in the presence of miR-302c, and miR-320a compared with the control mimic vector (Figure 3E). Thus, the candidate miRNAs (miR-302c, miR-320a, miR-542-3p, miR-641, miR-661 and miR-940) could regulate 5′-UTR of MICB by direct binding.

The possible mechanisms of candidate miRNAs that down-regulated at both 3′- and 5′-UTR were not the miBridge type but rather used its seeding region acting on target sequences separately on 3′-UTR and 5′-UTR. To investigate the possible mechanism of 3′-UTR and 5′-UTR on MICB regulation, two types of mutant reporter constructs were created. One type contained a mutated binding site of known miRNAs on 3′-UTR and mutated binding sites of only candidate miRNAs on the 5′-UTR (pMut_Known _3U_M5U) and another contained mutated both binding sites of known and candidate miRNAs on 3′-UTR and mutated binding sites of only candidate miRNAs on 5′-UTR (pMut_Known+6 Novel miRNAs_3U_M5U). The luciferase activities were slightly increased (5%) in pMut_Known_3U_M5U vector and strongly increased (40%) in pMut_Known+6 Novel miRNAs_3U_M5U vector compared with pMICB_3U_5U, constructs containing both wild-type 3′- and 5′-UTR (Figure 3F). This is suggesting that the repression was not the miBridge model. The candidate miRNAs did not use 3′-end interacted at 5′-UTR and turn 5′-end interacted at 3′-UTR but independently bound at their target sequences on 3′-UTR and 5′-UTR. This is because the luciferase activity was little recovered only when binding sites of candidate miRNAs were mutated on the 5′-UTR but not on the 3′-UTR. On the other hand, the luciferase activity was recovered up to 80% when the mutated binding site of candidate miRNAs was present on both 3′-and 5′-UTR (Figure 3F). Thus, the 5′-UTR contribution on regulation of MICB was less than that of 3′-UTR. To confirm whether candidate miRNAs regulated MICB expression via interaction between seed region with the 3′-UTR and the 5′-UTR of MICB, we overexpressed the candidate miRNAs and co-transfected with constructs containing both wild-type 3′- and 5′-UTR (pMICB_3U_5U) into 239T cells. After overexpression, the mimic miRNAs were expressed higher than the endogenous miRNAs (Supplementary Figure S3D). The luciferase activity results were similar to those of the 3′-UTR experiments (Figure 3G). As expected, the effect of 3′-UTR over rules the effect of 5′-UTR in consistence with previous experiments.

### 3.5. Candidate miRNAs Control Cell Surface MICB Expressions

To demonstrate the miRNA regulation of MICB protein expression in cancer cell lines, two models were designed: (i) HeLa, a cervical cancer cell line with high expression of MICB was transfected with mimic miRNAs to repress MICB expression; (ii) KKU-214, a cholangiocarcinoma cell line, with low MICB expression was transfected with anti-sense against endogenous miRNAs to knock down miRNAs leading to increased MICB expression.

**Figure 4:**
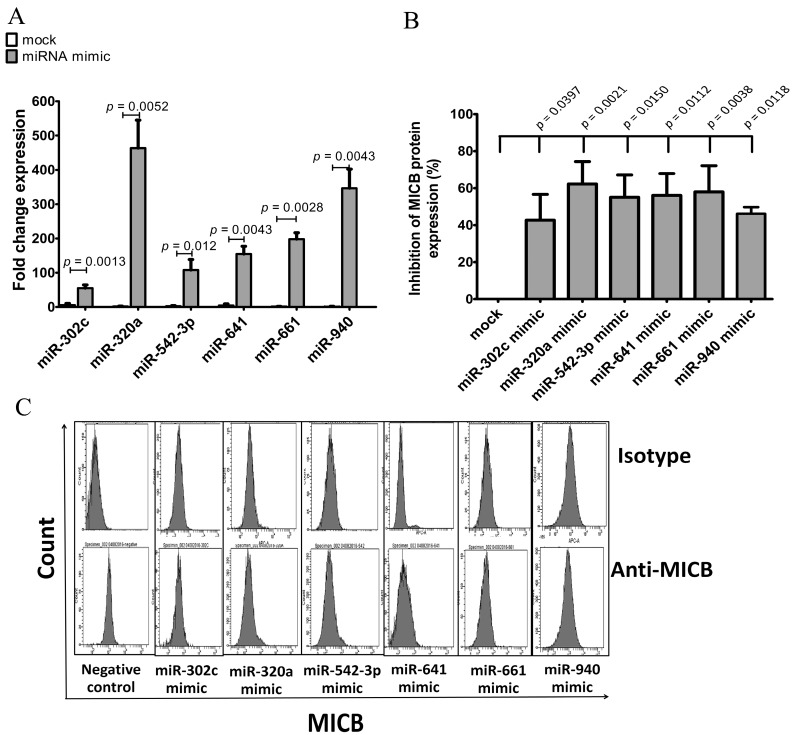
Inhibition of MICB expression on HeLa after transfection of miRNA mimics. (**A**) Fold change expression of miRNA compared to miRNA mimic control or mock (mock is transfected cells with irrelevant miRNA mimic). miRNAs were detected after transient transfection of 5 μg miRNA mimic control, miR-302c mimic, miR-320a mimic, miR-542-3p mimic, miR-641 mimic or miR-940 mimic into Hela cells. The expression levels were normalized to miR-16 expression. Fold change expression was calculated as: 2^−^^ΔΔCt^, where ΔΔCT = ΔCT (mimic miRNA transfected sample) − ΔCT (untransfected sample). (**B**) The expression of surface MICB was measured by flow cytometry after transfected with miRNA mimics into HeLa cells. The data shown in percentages of inhibition of MICB expression in mean fluorescence intensity (MFI) calculated from three experiments; (**C**) the histogram of flow cytometry is presented; the upper panel was stained with isotype control and the lower panel was stained with anti-MICB antibody. Resultsare depicted as mean ± S.E.M, *n* = 3 experiments.

Overexpression of candidate miRNAs in HeLa was performed by transfection with miRNA mimic vectors (miR-302c, miR-320a, miR542-3p, miR-641, miR-661 and miR-940) or control mimic vector. miRNAs and MICB protein expression levels were determined by qRT-PCR and flow cytometry, respectively. The overexpression experiments of miRNAs were successful with higher expression level of the mimic miRNAs than the endogenous miRNAs (Figure 4A). Similar to the reporter assay results, when we overexpressed miRNAs, MICB protein expression was significantly decreased on HeLa cell compared with mocked mimic (Figure 4B,C). These results indicated that overexpression of the candidate miRNAs (miR-302c, miR-320a, miR542-3p, miR-641, miR-661 and miR-940) could, indeed, reduce the surface expression of MICB protein. Finally, we concluded that not only MICB expression was regulated by miR-320a and miR-940 but could also be controlled by other candidate miRNAs such as miR-302c, miR-542-3p, miR-641 and miR-661. Albeit, the percentages of MICB protein expression was not so much decreased.

**Figure 5:**
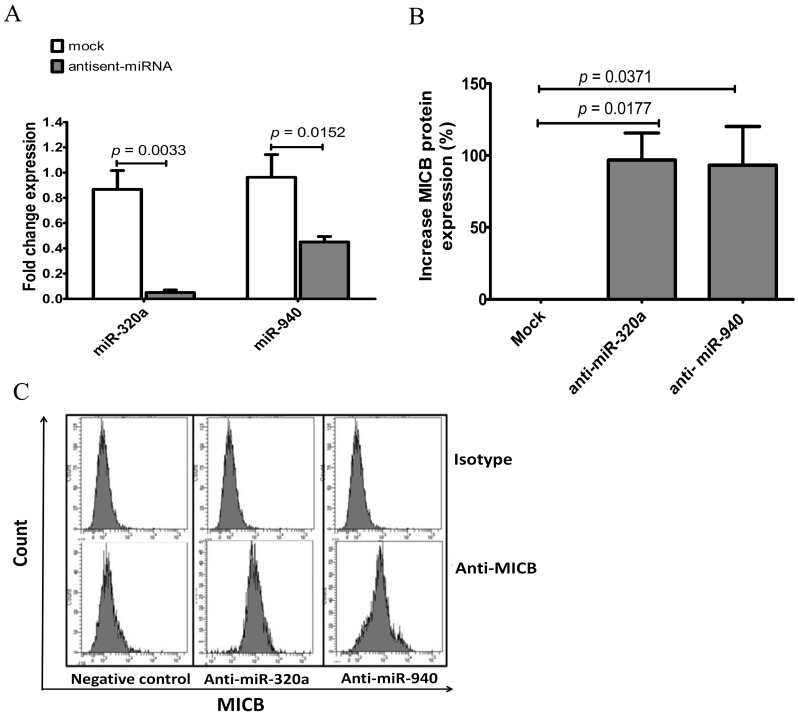
Increased MICB expression on KKU-214 cells after transfection with antisense miRNAs. (**A**) Fold change expression of miRNAs compared to the miRNA scramble control or mock (mock is transfected cells with irrelevant antisense-miRNA), miRNA expressions were presented after transient transfection of 500 nM miRNA scramble control (labelled as negative control in C), antisense-miR-320a or antisense-miR-940 into KKU-214 cells. The expression levels were normalized to miR-16. Fold change expression was calculated as: 2^−^^ΔΔCt^, where ΔΔCT = ΔCT (antisense-miRNA transfected sample) − ΔCT (untransfected sample); (**B**) The expression of surface MICB was measured by flow cytometry after transfected with miRNA scramble control, anti-miR320a or anti-miR940 into KKU-214 cells. The percentages of increasing MICB expressions based on the MFI calculated from three experiments; (**C**) Upper histogram was stained with isotype control and lower histogram was stained with anti-MICB antibody. Results are depicted as mean ± S.E.M, *n* = 3 experiments.

On the other hand, the role of miRNA candidates was confirmed using anti-sense RNAs to inhibit candidate miRNAs leading to increased MICB protein expression. As mentioned previously, miR-320a and miR-940 were expressed at high levels in several cell lines especially in KKU-214. Moreover, the exogenous miR-320a and miR-940 could also reduce luciferase activities (Figure 3C,E,G). KKU-214 cells were transfected with anti-sense miR-320a, anti-sense miR-940 or the scramble control. The miR-320a and miR-940 levels were reduced 90% and 55%, respectively, lower than the endogenous miRNAs (Figure 5A). Then, MICB protein expression levels were measured by flow cytometry. The surface expression of MICB was increased in the anti-miR- 320a and anti-miR-940 treated KKU-214 compared to the scramble controls (Figure 5B,C). Thus, we concluded that candidate miRNAs (miR-320a and miR-940) genuinely regulated MICB expression.

## 4. Discussion

Several studies reported that MICB expression was controlled by various miRNAs via binding at the 3′-UTR of MICB leading to repression of protein expression [16,17,31,32]. In this study, we have identified novel miRNAs regulating MICB expression via both3′-UTR and 5′-UTR sequences. Combined regulation effect of several miRNAs, especially at 3′-UTR, has been demonstrated emphasizing the need to identify all miRNAs regulating on a target gene.

Diverse computational algorithms could predict various miRNA targeting at 3′-UTR and 5′-UTR of MICB. However, we detected nine miRNAs that were all predicted by three different prediction programs (TargetScan, RNAhybrid and miBridge). Interestingly, all nine miRNAs are the miRNAs that have not been reported. The studies of Stern-Ginossa et al. reported that miR-20a, miR-93 and miR-106b could control MICA and MICB expressions [16] and recent study of Tsukerman et al. also showed that MICB expressions were regulated by miR-10b [31]. The correlation between protein and mRNA expression of MICB in cancer cell lines indicated inverted correlation between protein and mRNA levels. Moreover, in these cell lines various expressions of the candidate miRNAs were present. These findings suggest that regulation of MICB at post transcriptional level exists [9,33]. In addition, our study suggests that multiple miRNAs could have a combined effect in gene regulation. This would applicable for therapeutic approach targeting miRNAs in cancer.

The study of miRNA expression pattern in intrahepatic cholangiocarcinoma (ICC) by Chen et al. [34], indicated that miR-320 was down expressed in ICC. Plieskatt et al. [35] found that miR-320b in plasma was one of the 8 miRNAs which were a signature of ICC. In addition, miR-320a was also displaying high expression in plasma but low expression in the ICC tissues. They postulated that ICC tumor sheded miRNA into the blood stream. These reflect the possible different functions of miRNAs in tissues and circulating miRNA in peripheral blood. Our results showed that miR-320 cluster (miR-320a, miR-320b, miR-320c and miR-320d) could down regulated MICB expression in human cancer cells, especially miR-320a that was highly expressed in a CCA cell line (KKU-214). Expressions of NKG2D ligands on cancer cells are stage dependent, expression at the early development would benefit to the patients but chronic expression of the ligands leading to immune evasion [36]. Nevertheless, our study could support the role of miR-320 in cancer.

Most studies aimed to investigate regulation of gene expression by miRNAs via binding at 3′-UTR of the target genes [19,37,38] but our study also focused on 5′-UTR. Our functional studies of MICB gene regulation by using dual luciferase reporter assay, overexpression of miRNAs and using anti-sense miRNAs reveal that MICB expression were repressed via interaction between miRNAs with 3′- and 5′-UTR of MICB. However, it was not via the miBridge model which a single miRNA had combinatory interactions with both UTRs of an mRNA [12]. We did not find the combined effect of 3′- and 5′-UTR regulation. The 3′-UTR contribution on regulation of MICB was more than that of 5′-UTR. However, the combinatory effect of both 3′- and 5′-UTR was not found to enhance MICB regulation more than that of 3′-UTR alone. We postulated that the whole miRNA interacted with target sequences at 3′-UTR and 5′-UTR of MICB independently instead of one halve of miRNA bound at 3′-UTR and another halve at 5′-UTR. A structural analysis would be required to confirm this observation.

In previous study, Deepak et al. showed that miR-520b could inhibit MICA gene expression by binding on the promoter region and probably regulating transcription factors that important for MICA expression [17]. Similar to our finding, MICB gene expressions were regulated via candidate miRNAs at the 5′-UTR sequence of MICB. The functions of miRNAs to regulate gene expression at 5′-UTR are largely unknown. We speculate that miRNAs could suppress MICB expression by interfering the transcription binding site at 5′-UTR. Using a computational prediction, we have found that some candidate miRNAs binding sites were on or close to transcription factor binding sites such as miR-641 and miR-661 having binding sites on the activator protein 1 (AP-1) which regulates gene expression in response to cellular stress, bacterial and viral infections [39]. miR-542-3p and miR-940 have binding sites on the core promoter, initiator element (Inr) that has function similar to TATA in facilitating TATA binding protein or the transcription factor II D (TFIID) [40]. The binding of TFIID to the TATA box or Inr in the core promoter region would recruit other factors required for RNA polymerase II to start transcription [41], suggesting that these miRNAs may regulate MICB via 5′-UTR by interfering the binding of transcription factors to transcription factor-binding sites or responsive element essential for MICB expression. This new role of miRNA to regulate at the transcriptional level awaits further investigations.

Recently, Su et al. reported that miR-940 expressions were upregulated in cervical cancer tissue samples and cell lines and induced cervical cancer cell growth, proliferation and cell cycle arrest in vitro as well as tumor formation in vivo via suppression of p27 and PTEN expressions [42]. Loosing of this gene was involved in the modulation of tumor cell growth and proliferation. It is consistent to our study that miR-940 was highly expressed in cervical cancer cell lines and cloud down regulate MICB expression in cancer cells leading to immune surveillance escape and surviving in the host. This may be a therapeutic target for cervical cancer treatment in the future by inhibition of candidate miRNAs including miR-940 using antisense oligonucleotide to block endogenous miRNA function for enhancement of immune cell function and inhibition of tumor cell growth and proliferation.

In conclusion, we have identified novel miRNAs regulating MICB expression by targeting at both 3′- and 5′-UTR. In addition, this study has also identified a new role of miRNAs in regulating MICB expression via 5′-UTR and provides information for further study on novel mechanisms of cellular miRNAs in regulation at the transcriptional level applicable for cancer immunotherapy.

## Figures and Tables

**Table 1:** List of plasmid constructs.

Plasmid Names	Information
pMICB_3U	Wild type 3′-UTR of MICB
pMICB_5U	Wild type 5′-UTR of MICB
pMICB_3U_5U	Wild type 3′- and 5′-UTR of MICB
pMut_Known+6 Novel miRNAs	mutated both known and novel miRNAs binding sites at 3′-UTR (no inserted 5′-UTR)
pMut_Known miRNAs	mutated only known miRNAs binding sites 3′-UTR (no inserted 5′-UTR)
pMut_320a	mutated binding site of only miR-320a 5′-UTR (no inserted 3′-UTR)
pMut_miR320a+5 Novel miRNAs	mutated binding sites of all candidate miRNAs 5′-UTR (no inserted 3′-UTR)
pMut_Known_3U_M5U	mutated binding sites of known miRNAs on 3′-UTR and mutated binding sites of only candidate miRNAs on 5′-UTR
pMut_Known+6 Novel miRNAs_3U_M5U	mutated both binding sites of known and candidate miRNAs on 3′-UTR and mutated binding sites of only candidate miRNAs on 5′-UTR

**Table 2:** Novel candidate microRNAs (miRNAs) binding sites at 5′-untranslated region (UTR) and 3′-UTR of MHC class I chain related protein B (MICB).

miRNA	5′-UTR			3-′UTR		
	Seed Sequence	Binding Sequence	Free Energy (Kcal/mol)	Seed Sequence	Binding Sequence	Free Energy (Kcal/mol)
miR-302c	UUCAGUGG	^77^UCGCUGAG ^a^	−21.7	AAGUGC	^736^GCACUU ^a,b,c^	−20.3
miR-320a	UGAGAGGG	^51^UCUUCUCA ^a^	−24.4	AAAGCU	^1103^AGCUUU ^a,b,c^	−24.7
	AAGCUGG	^60^CCGGUUU ^b^	−25.8			
miR-320b	UGAGAGGG	^51^UCUUCUCA ^a^	−24.4	AAAGCU	^1103^AGCUUU ^a,b,c^	−24.7
	AAGCUGG	^60^CCGGUUU ^b^	−25.8			
miR-320c	UGAGAGGG	^51^UCUUCUCA ^a^	−21.0	AAAGCU	^1103^AGCUUU ^a,b,c^	−24
	AAGCUGG	^60^CCGGUUU ^b^	−22.4			
miR-320d	UGAGAGGA	^51^UCUUCUCA ^a^	−21.3	AAAGCU	^1103^AGCUUU ^a,b,c^	−24.3
	AAGCUGG	^60^CCGGUUU ^b^	−22.7			
miR-542-3p	UAACUGAA	^69^UUCAGUUG ^a^	−18.1	GUGACA	^671^UGUCAC ^a,b,c^	−21.7
miR-641	AGUCACCU	^30^AGGUGACU ^a^	−24.4	AAGACA	^526^AUGUCU ^a,b,c^	−16.3
	AAGAC	^51^GUCUU ^b^	−21.3			
miR-661	GCCUGCGC	^26^GCGCAGGU ^a^	−30.8	GCCUGG	^677^CCAGGC ^a,b,c^	−26.8
	GCCUGGG	^56^CUCACCGGU ^b^	−30.5			
miR-940	CCCCGCUC	^19^GAGCGGGG ^a^	−27.6	AGGCAG	^32,881^CUGCCU ^a,b,c^	−28.5
	GGCAGGGCC	^75^GGCCACUCCU ^b^	−26.3			

^a^ analyzed by miBridge; ^b^ analyzed by RNA hybrid; ^c^ analyzed by TargetScan.

## Supplementary Materials

The following are available online at www.mdpi.com/2073-4425/8/9/213/s1. Figure S1: The mRNA expression level of MICB in human cancer cells. Figure S2: The surface expression level of MICB in human cancer cells. Figure S3: Relative expression of candidate miRNAs and fold change expression of overexpression miRNA in 293T cell line. Table S1: Specific primers for 3’ and 5’-UTR amplifications. Table S2: Primers for miRNAs amplification. Table S3: Primers for point PCR mutations of 3’ -UTR of MICB., Table S4: Primers for point PCR mutations of 5’ -UTR of MICB.
