# Supplementary material for: 5′-UTR and 3′-UTR Regulation of MICB Expression in Human Cancer Cells by Novel microRNAs
Source: Genes (Basel). 2017 Aug 29;8(9):213. doi: 10.3390/genes8090213 (PMC5615347; doi:10.3390/genes8090213)
Supplement: Supplementary file 1 [file genes-08-00213-s001.pdf]

Supplementary Figure S1.

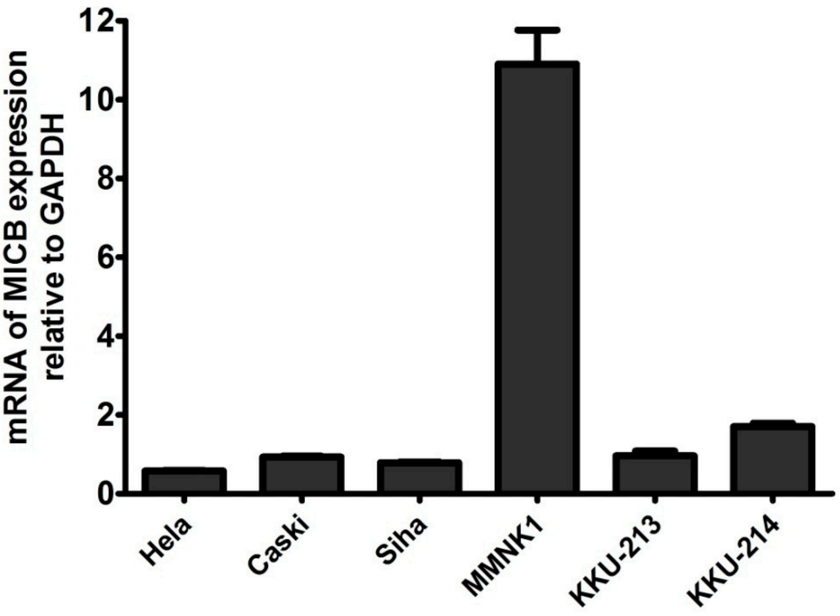

Supplementary Figure S1. The mRNA expression level of MICB in human cancer cells. Cervical cancer cells (HeLa, Caski and SiHa), cholangiocarcinoma (CCA) cells (KKU-213 and KKU-214 and immortalized cholangiocyte (MMNK1) were measured for mRNA expression levels of MICB using qRT-PCR and normalized with GADPH. Results are mean ± SEM, n=3 experiments.

Supplementary Figure S2.

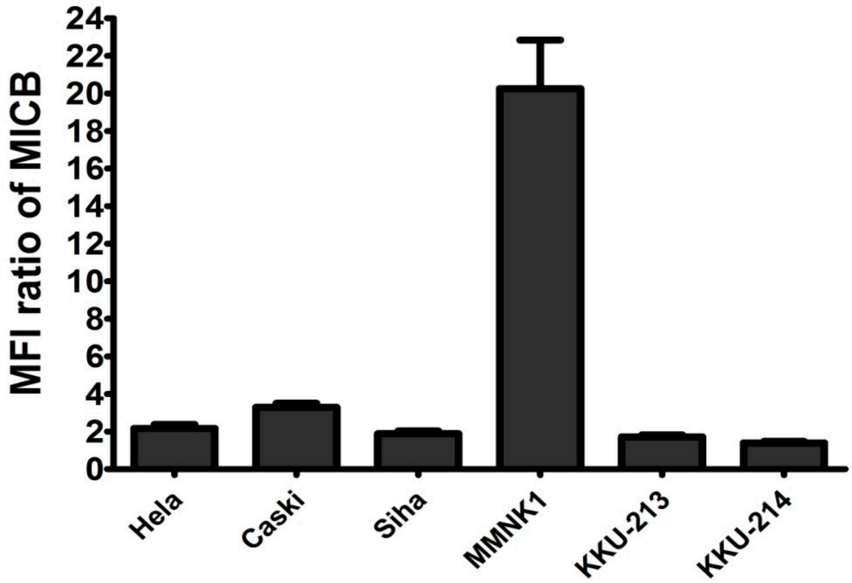

Supplementary Figure S2. The surface expression level of MICB in human cancer cells. Cervical cancer cells (HeLa, Caski and SiHa), cholangiocarcinoma (CCA) cells (KKU-213 and KKU-214) and immortalized cholangiocyte (MMNK1) were measured for protein surface expression levels of MICB using flow cytometry. The MICB expression level was determined by the mean fluorescence intensity (MFI) calculated by the MFI of cells stained with an anti-MICB Ab divided by the MFI of cells stained with the isotype antibody. Results are mean ± SEM, n=3 experiments.

Supplementary Figure S3.

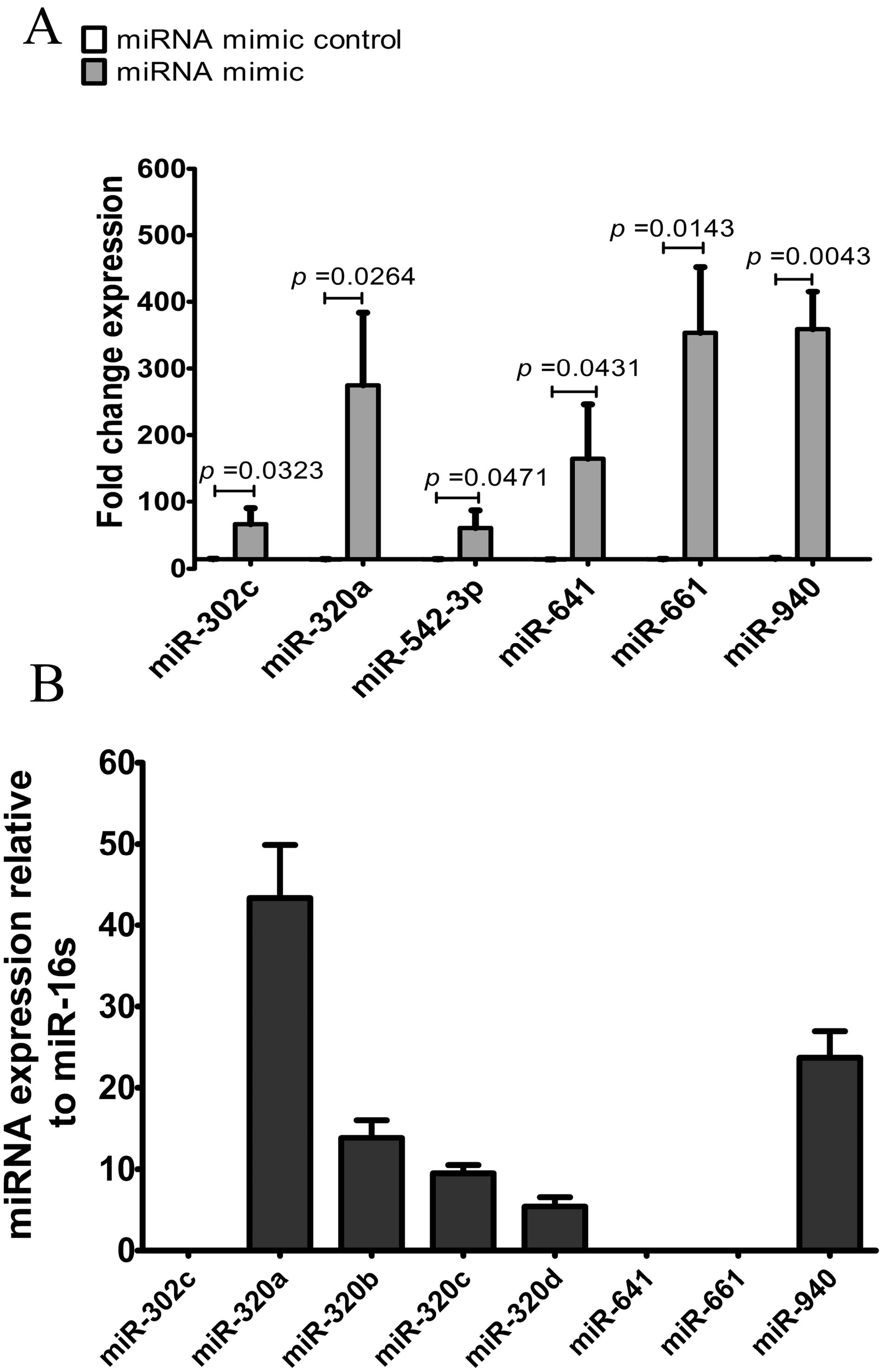

C

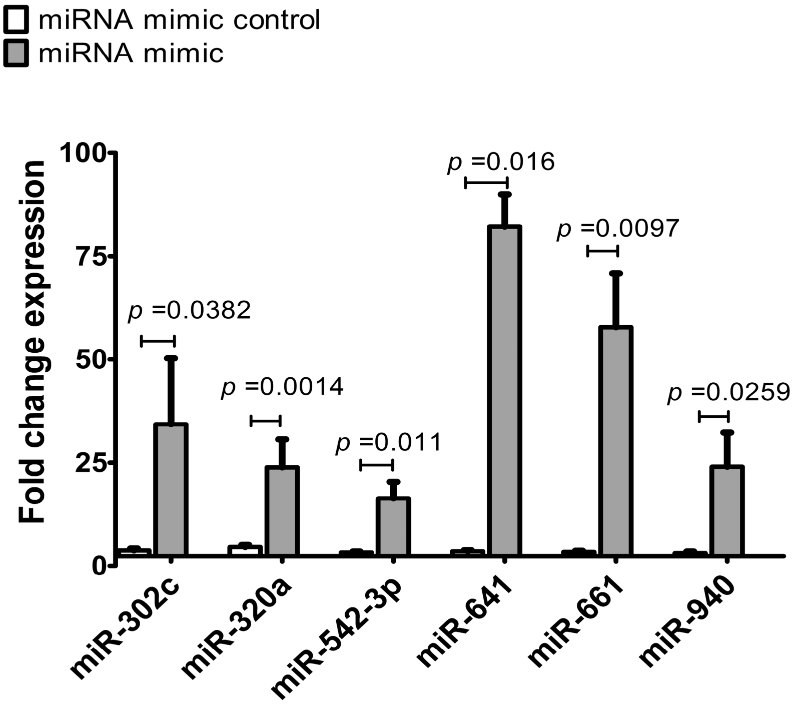

D

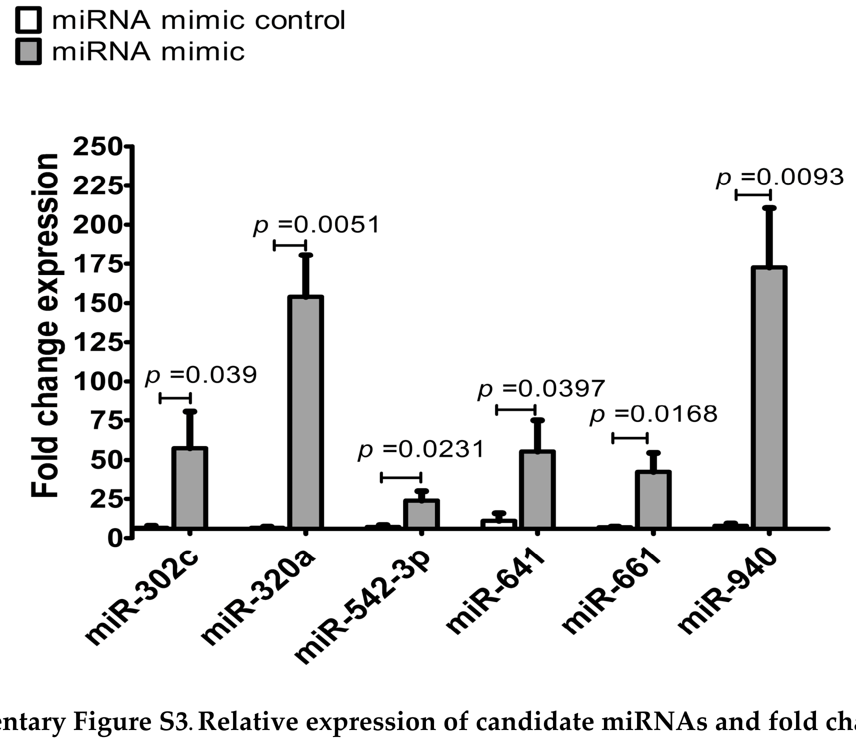

Supplementary Figure S3. Relative expression of candidate miRNAs and fold change expression of overexpression miRNA in 293T cell line. (A) Fold change expression of miRNA compared to mimic miRNA control in overexpression of mimic miRNA and co-transfection with reporter construct containing wild-type 3-UTR (pMICB_3U) (B) Endogenous expression levels of nine candidate miRNAs were measured in 293T cell lines by qRT-PCR. The expression levels were normalized to miR-16. The results were mean±S.E.M. Relative expression was calculated as: $2^{\Delta Ct}$, where $\Delta CT= CT_{givenmiRNA}-CT_{miR.16}$. . (C) Fold change expression of miRNA compared to mimic miRNA control in overexpression of mimic miRNA and co-transfection with reporter construct containing wild-type 5-UTR (pMICB_5U) . (D) Fold change expression of miRNA compared to mimic miRNA control in overexpression of mimic miRNA and co-transfection with reporter construct containing both wild-type 3′ and 5′ -UTR (pMICB_3U_5U) . Fold change expression was calculated as: $2^{-\Delta\Delta Ct}$, where $\Delta\Delta CT=\Delta CT$ (mimic miRNA transfected sample)- ΔCT (untransfected sample).

Supplementary materials and methods

Supplementary Table S1. Specific primers for 3' and 5' -UTR amplification

Gene names	Primer names	Sequences from 5' to 3'
3'UTR MICB	F_3'UTR_MICB	5'-TTCGAGGATCCACTCTACAGCCAGGCGGCCAGGATTC-3'
	R_ 3'UTR_MICB	5'-GCGCCGCGGCCGCTCTATTTAAAGAGTAAACATTTACT-3'
5'UTR MICB	F_5'UTR_MICB	5'-CGCGCGCTAGCACTGGATAAGCGGTCGCTGA-3'
	R_5'UTR_MICB	5'-CGCGCAAGCTTGGCCCCTACGTCGCCACCTT-3'

Supplementary table S2. Primers for miRNA amplification

miRNA names	Sequences from 5' to 3'	
	**Forward primers**	**Stem loop primers**
miR-302c	5'-GGCCGTAAGTGCTTCCATGTTT-3'	5'-GTCGTATCCAGTGCAGGGTCCGAGGTATTCGCACTGGATACGACCCACTG -3'
miR-320a	5'-CGCCGAAAAGCTGGGTTGAGA-3'	5'-GTCGTATCCAGTGCAGGGTCCGAGGTATTCGCACTGGATACGACTCGCCC-3'
miR-320b	5'-CGGGCGAAAAGCTGGGTTGAGA-3'	5'-GTCGTATCCAGTGCAGGGTCCGAGGTATTCGCACTGGATACGACTTGCCC-3'
miR-320c	5'-GCGGCGGAAAAGCTGGGTTGA-3'	5'-GTCGTATCCAGTGCAGGGTCCGAGGTATTCGCACTGGATACGACACCCTC-3'
miR-320d	5'-GCGGCGAAAAGCTGGGTTG-3'	5'-GTCGTATCCAGTGCAGGGTCCGAGGTATTCGCACTGGATACGACTCCTCT-3'
miR-542-3p	5'-GGCGGTGTGACAGATTGATAA-3'	5'-GTCGTATCCAGTGCAGGGTCCGAGGTATTCGCACTGGATACGACTTTCAG-3'
miR-641	5'-GCCGAAAGACATAGGATAGAGT-3'	5'-GTCGTATCCAGTGCAGGGTCCGAGGTATTCGCACTGGATACGACGAGGTG-3'
miR-661	5'-CGTGCCTGGGTCTCTGGCCT-3'	5'-GTCGTATCCAGTGCAGGGTCCGAGGTATTCGCACTGGATACGACACGCGC-3'
miR-940	5'-GTGGAAGGCAGGGCCCCCG-3'	5'-GTCGTATCCAGTGCAGGGTCCGAGGTATTCGCACTGGATACGACGGGGAG-3'
	**Universal reverse primer**	
	5'-GTGCAGGGTCCGAGGT-3'	

Supplementary Table S3. Primers for point PCR mutations of 3' -UTR of MICB

Primer names	Sequences from 5' to 3'
F_3'UTR_MICB	5'-TTCGAGGATCCACTCTACAGCCAGGCGGCCAGGATTC-3'
R_49-56_3U	5'-AACAGAGGGAATCTGCTGGTGA -3'
F_49-56_3U	5'-TCACCAGCAGATTCCCTCTGTT -3'
R_734-742_3U	5'-GAGGTACGAGATC TGCTTGAAC -3'
F_734-742_3U	5'-GTTCAAGCAGATCTCGTACCTC -3'
R_1110_3U	5'-GACAAGAAATATGGTCCTGG -3'
F_1110_3U	5'-CCAGGACCATATTTCTTGTC- 3'
R_ 3'UTR_MICB	5'-GCGCCGCGGCCGCTCTATTTAAAGAGTAAACATTTACGT- 3'
F_3'UTR_MICB	5'-TTCGAGGATCCACTCTACAGCCAGGCGGCCAGGATTC- 3'
R_28-41_3U	5'-GAGATCCACTGAGGGAGTT- 3'
F_28-41_3U	5'-AACTCCCTCAGTGGATCTC- 3'
R_517-538_3U	5'-TAAAGAGAACTGCAAAAGAGT -3'
F_517-538_3U	5'-ACTCTTTTGCAGTTCTCTTTA- 3'
R_ 665-686_3U	5'-ACTGCAGCCTTCTGGACAGAGTGA -3'
F_665-686_3U	5'-TCACTCTGTCCAGAAGGCTGCAGT- 3'
R_873-890_3U	5'-ATGCCAAGCTGGGCAGATC- 3'
F_873-890_3U	5'-GATCTGCCCAGCTTGGCAT-3'
R_923-940_3U	5'-TAAAATAGCTGGGCACGGT-3'
F_923-940_3U	5'-ACCGTGCCCAGCTATTTTA-3'
R_ 3'UTR_MICB	5'GCGCCGCGGCCGCTCTATTTAAAGAGTAAACATTTACGGT- 3'

Supplementary Table S4. Primers for point PCR mutations of 5' -UTR of MICB

Primer names	Sequences from 5' to 3'
F_5UTR_MICB	5'- CGCGCGCTAGCACTGGATAAGCGGTCGCTGA-3'
R_55_5U	5'-AACCCGTGAGGTGACCCCGT-3'
F_55_5U	5'-ACGGGGTCACCTCACGGGTT- 3'
R_5UTR_MICB	5'- CGCGCAAGCTTGGCCCCTACGTCGCCACCTT- 3'
F_1-26_5U	5'-CGCGCGCTAGCACTGGATAAGCGGTCGCTGACTGGGG -3'
R_64-88_5U	5'-CTCAGCAGTGGTGTGCTGAATGAAAC-3'
F_64-88_5U	5'-GTTTCATTCAGCACACCACTGCTGAG-3'
R_5UTR_MICB	5'-CGCGCAAGCTTGGCCCCTACGTCGCCACCTT-3'
